# Temporal discrepancies in “rapid” HIV testing: explaining misdiagnoses at the point-of-care in Zimbabwe

**DOI:** 10.1186/s12879-022-07972-5

**Published:** 2023-01-06

**Authors:** Morten Skovdal, Frederik Jacob Brainin Jensen, Rufurwokuda Maswera, Nadine Beckmann, Constance Nyamukapa, Simon Gregson

**Affiliations:** 1 Department of Public Health, University of Copenhagen, Øster Farimagsgade 5, 1014 Copenhagen, Denmark; 2 Manicaland Centre for Public Health Research, Biomedical Research and Training Institute, Harare, Zimbabwe; 3 Department of Global Health and Development, London School of Hygiene and Tropical Medicine, London, United Kingdom; 4 Department of Infectious Disease Epidemiology, Imperial College London, London, United Kingdom

**Keywords:** HIV testing, Rapid tests, Point-of-care test, Testing errors, Zimbabwe

## Abstract

**Background:**

Rapid diagnostic tests have revolutionized the HIV response in low resource and high HIV prevalence settings. However, disconcerting levels of misdiagnosis at the point-of-care call for research into their root causes. As rapid HIV tests are technologies that cross borders and have inscribed within them assumptions about the context of implementation, we set out to explore the (mis)match between intended and actual HIV testing practices in Zimbabwe.

**Methods:**

We examined actual HIV testing practices through participant observations in four health facilities and interviews with 28 rapid HIV testers. As time was identified as a key sphere of influence in thematic analyses of the qualitative data, a further layer of analysis juxtaposed intended (as scripted in operating procedures) and actual HIV testing practices from a temporal perspective.

**Results:**

We uncover substantial discrepancies between the temporal flows assumed and inscribed into rapid HIV test kits (their intended use) and those presented by the high frequency testing and low resource and staffing realities of healthcare settings in Zimbabwe. Aside from pointing to temporal root causes of misdiagnosis, such as the premature reading of test results, our findings indicate that the rapidity of rapid diagnostic technologies is contingent on a slow, steady, and controlled environment. This not only adds a different dimension to the meaning of “rapid” HIV testing, but suggests that errors are embedded in the design of the diagnostic tests and testing strategies from the outset, by inscribing unrealistic assumptions about the context within which they used.

**Conclusion:**

Temporal analyses can usefully uncover difficulties in attuning rapid diagnostic test technologies to local contexts. Such insight can help explain potential misdiagnosis ‘crisis points’ in point-of-care testing, and the need for public health initiatives to identify and challenge the underlying temporal root causes of misdiagnosis.

## Introduction

Rapid diagnostic tests for the human immunodeficiency virus (HIV) have been designed to provide test results within short timeframes, enabling HIV testers at the point-of-care to make a quick diagnosis, initiate early treatment, and provide immediate post-test counselling [1]. Rapid HIV tests thus shorten the diagnostic waiting time significantly, reducing loss to follow-up of patients, a common challenge of more traditional laboratory-based enzyme-linked immunosorbent assay (ELISA) testing [2]. Rapid HIV tests address important time issues, and have played a crucial role in accelerating the 95-95-95 goals [3]. Whilst rapid HIV tests have offered important speed and reach to HIV testing in general, disconcerting levels of misdiagnosis have been reported [4]. As time-constraints have been identified as a key contributor to suboptimal point-of-care testing [5], we ask: with what speed should HIV testers conduct a rapid HIV test? How does this speed match the reality that HIV testers meet in the clinics? We explore these questions with a focus on the temporalities of labelling, reading and recording rapid HIV tests. In doing so we respond to previous work that notes time (or lack thereof) as a contributing factor to misdiagnosis, and present a fine-grained and theory-informed analysis of the temporalities rapid HIV testing.

Multiple studies report elevated levels of misdiagnosis in rapid testing of HIV. A systematic review by Johnson and colleagues [4] of studies reporting on misdiagnosis in rapid HIV testing found average levels of false-negative and false-positive HIV test results to be 0.4% and 3.1% respectively. The review also found that 0.1–6.6% of the people who had been enrolled in HIV care and/or were initiated onto antiretroviral therapy (ART) were in fact HIV-negative. Misdiagnosis has also been observed in Zimbabwe. Recently, a thorough investigation of HIV misclassification errors in Zimbabwe found a very high level of false-negative results (10%) and a significantly lower level of false-positive results (0.1%) [6]. The study also found a significant number of thought-to-be-positive people (3.5%) on antiretroviral therapy who in fact were HIV negative [6]. Although these levels of misdiagnosis are relatively low, the absolute numbers of misdiagnosed individuals raise serious concerns, not least when the human [7, 8] and financial [9] costs associated with misdiagnosed HIV infection are taken into consideration. The concerns underscore the need to unpack potential causes of misdiagnosis.

A number of factors and practices may contribute to misdiagnosis, including user errors [10], clerical and technical errors [11], poor management and supervision [12], interpretation of weak reactive test results [13], and the use of suboptimal testing strategies and algorithms [7]. In Zimbabwe, we have observed a lack of confidence amongst HIV testers in using certain diagnostic test-kits, explained by regular changes to testing algorithms and inadequate training [14]. We also found social interactions, from power struggles between HIV testers to uncomfortable encounters between HIV testers and their clients, to challenge the testing process. Finally, we found difficult work conditions to contribute to HIV testers getting distracted and experiencing time-constraints [14], an issue we explore further in this article.

In a review of barriers to adoption and scale-up of point-of-care tests (not only for HIV) in low and middle-income countries, Pai et al. [5] find time to be of particular concern. They find the combination of staff shortages and high patient volumes to contribute to time-constraints that occasionally result in suboptimal testing practices. This may explain observations made in South Africa, where only 1 of 10 observed testing practices were accurately timed [15]. Given the dearth of research exploring the role of time and temporal factors in misdiagnosis, we set out to investigate temporal discrepancies between intended and actual testing practices.

### The temporalities of scripted rapid HIV testing

Two interrelated concepts steer our framing and analysis. The first concept, “scripts”, heightens our attention to the intended HIV testing practice. The concept enables us to understand how the rapid HIV test-kits—as technical objects—have inscribed within their design particular visions and assumptions about the relationships that exist between a technical object, its users and the accompanying context of implementation [16, 17]. For rapid HIV tests, these inscriptions are elucidated in protocols, manuals, or training materials on how to use the test kits efficaciously, minimizing diagnostic errors. This encourages us to examine the relationship between these scripted and intended HIV testing steps and what happens in real life. Instructions on how to use the test-kits may come directly from the designers and manufacturers, or may be appropriated, or tinkered with, by local stakeholders, such as national health ministries, as they formulate documents and guidelines to help rapid HIV testers in their respective contexts. Table 1 exemplifies step-by-step instructions given to HIV testers in Zimbabwe for two commonly used test-kits, alluding to the speed of the rapid HIV tests. The instructions are ordinarily in a poster format and include a photo for each step. Although these scripted steps are the focus of this article, they form part of a broader set of procedures that precede and follow, including steps related to storage, hygiene, and quality assurance, testing strategies [14, 18], pre- and post-counselling.

**Table 1:** Scripted steps for using rapid HIV test-kits

Step	Description	
	Test-kit 1	Test-kit 2
1	Collect all necessary supplies.	Collect all necessary supplies.
2	Review and record the master kit lot number and expiry date.	Remove device from packaging and label with client identification number.
3	Use 1 strip per client. Label test strip with client identification number.	Collect approximately 5 µl of specimen using a new disposable loop.
4	Pull off the protective foil cover.	Touch loop to center of sample well to transfer blood specimen to the well.
5	Collect 50 µl of specimen using a capillary tube. Fill sample between the marked fill line.	Add 3 drops of buffer, holding vial vertically over the sample well.
6	Apply sample to absorbent pad on the strip. For whole blood, wait for blood to absorb on pad.	Wait for 15 minutes (no longer than 20 minutes) before reading the results
7	For whole blood only add 1 drop of chaser buffer to the specimen pad.	Read and record the results and other pertinent info on the worksheet.
8	Wait 15 minutes (no longer than 60 minutes) before reading the results.	
9	Read and record the results and other pertinent info on the worksheet.	

The second concept, temporality, helps us compare and contrast scripted and everyday HIV testing practices from a time perspective. Here we draw on Barbara Adam’s [19] time-scape conceptualization of temporality. She outlines different temporal elements, which encourage us to explore: the *timeframe* of HIV testing (e.g., how long it takes to execute the steps outline in Table 1); the *timing* of HIV testing steps (including their synchronicity with the actions of HIV testers); who and what establishes the *tempo* of HIV testing; how *momentum* in HIV testing are achieved or come to a halt; the *sequence* or order of steps in using rapid HIV tests within a given timeframe; and the *frequency* of HIV testing steps that need to be performed within a given timeframe. Adam [19] posits that none of the above temporal elements operate alone. They are all mutually dependent and shape each other. Together with the concept of ‘scripts’ these temporal elements help us explore the time factors that shape the pace of rapid HIV testing, and whether and how the timings inscribed into the steps of using rapid HIV test-kits are compatible (or otherwise) with the timings and flows of everyday HIV testing practices.

## Methods

This qualitative investigation was part of a larger mixed-methods study, which also quantitatively examined the causes and consequences of misclassification errors in rapid HIV test algorithms ("the misclassification study"). The study was approved by the Medical Research Council of Zimbabwe (MRCZ/A/1865) and Imperial College London ethical review boards (15IC2797). Approvals were obtained on the condition that study participants gave their informed, written consent, and that anonymity was ensured. Pseudonyms have therefore been used throughout.

### Study location and participation

Data for this study was generated from HIV testers who were sampled from 14 health facilities across six districts in Zimbabwe: Chipinge, Gutu, Buhera, Harare, Mudzi and Rushinga. The health facilities were sampled from a national HIV surveillance survey of 62 antenatal clinics across Zimbabwe. A requirement of the study was that the health centres should represent a mixture of district hospitals and local clinics that had either performed reasonably well or poorly when it came to average rates of misdiagnosis. Also geography and logistics were considered when selecting the health facilities. Once the facilities had been selected, we invited the Matron or Sister from each facility to recruit two rapid HIV testers for interviews. Twenty-eight HIV testers agreed to participate. None of the invited HIV testers declined participation. Most participants were female (n = 20), worked in low performing facilities (n = 22) and district hospitals (n = 16), as opposed to in a local clinic. Most of the testers were employed as HIV counsellors (n = 17), whilst 10 worked as registered nurses and one participant worked as a lab technician. All HIV testers received a t-shirt as compensation for their time and participation in the study. In addition to the interviews, we spent time in four diverse health facilities, carrying out week-long participant observation in each of these facilities.

### Data collection and analysis

Data were collected between April and August 2018. The interviews were conducted by four local, experienced, and Shona-speaking qualitative researchers who were trained on the study objectives and procedures. The interviews followed a semi-structured topic guide, covering themes such as when and how rapid HIV tests were introduced in their clinic, experiences of stocking and administering rapid HIV tests, the quality and user-friendliness of the different rapid HIV tests, diagnostic errors, and their recommendations for improving rapid HIV testing. None of the questions included in the topic guide focused on the temporal aspects of rapid HIV testing. The interviews lasted between 45 and 60 min, were digitally recorded, transcribed and translated into English. The participant observations were conducted over a 4-week period. Observations were being carried out from 9 am to 4 pm, Monday through to Saturday in each of the four health facilities. This amounted to 24 observational visits. The observation guides focused on testing practices and paid attention to actors, objects, communication, and affordance as well as the temporal context of rapid HIV testing. Testing practices were observed in various departments, including the antenatal clinic, the HIV testing and counselling department, and the opportunistic infection/antiretroviral treatment department. In each health care facility, different rapid testers, who had received training in rapid testing, were observed.

All interview and observation data were imported into NVivo 12 for coding and thematic network analysis, following the steps of Attride-Stirling [20]. Inductive and in-depth coding of 10–12 transcripts was done by Skovdal (first author), Jensen (second author) and Beckmann (fourth author) independently from one another. Coding frameworks were compared, and a single coding framework was applied to the remaining dataset. Jensen proceeded with a more fine-grained analysis of his 12 interviews, devising and drawing on the conceptual framework to explore and juxtapose the temporalities of intended and actual rapid HIV testing. This resulted in 24 basic themes and six organizing themes. As we cannot report on the full analysis in this article, we focus on themes highlighting discrepancies between intended and actual testing practices. This condensation resulted in three organizing themes (detailed by 10 basic themes), which structure the presentation of our findings (see Table 2).

**Table 2:** Thematic network of emerging findings

Emerging themes (basic themes)	Temporal discrepancies (organizing themes)	Overarching story (global theme)
Clinics and hospitals experience staff shortages and high client volumes	1. High *frequency* testing affects correct labelling	Temporal factors shaping a discrepant flow of labelling, reading, and recording rapid HIV test results
Multiple clients are consulted at a time due to high client volumes to high frequency (and occasional repeat) testing		
High frequencies of clients cause confusion which leads to incorrect labelling		
Proper timing is essential to assess test results inside the valid results interval (VRI)	2. Mis*timing* the reading of test results	
Due to inadequate timekeeping practices, HIV testers read and report invalid tests		
Stress and pressure lead to inadequate timing of test results		
Timing specifications differ between RDTs		
It is easier to subscribe to timing practices if one client is tested at a time		
Recording results in multiple registers is considered time-consuming	3. Recording is time-consuming, but prioritized, affecting *sequential* steps	
Time-constraints, coupled with honouring recording requirements, affects counselling practices		

## Results

We identified three temporal discrepancies in labelling, reading, and recording rapid HIV test results, which may contribute to diagnostic misclassification errors.

### High *frequency* testing affects correct labelling

While the standard operating procedures for the rapid HIV test kits used in Zimbabwe do not stipulate any limits to how many tests a single person can administer at a time, prescriptions around the different steps involved (see Table 1) put a natural limit to how many tests a person can handle at the same time. Indeed, one participant mentioned that during a training on test kits it was emphasized that testing multiple clients at the same time is problematic, simply because it may lead to a mix-up.

What we were taught when we did our training was that we should deal with one client at a time and issue them their results rather than testing many people at a time. In such a case you can give someone a wrong test result. Karen, age 45, Avuma District Hospital.

Reflecting this, and when asked about their testing practices, participants often provided desirable accounts, explaining how they test one client at a time, so that they can carefully label the test-kits:

I will be dealing with one patient at a time so when they come for testing, I deal with them and everything about their results; and the moment that I put their blood sample on the test kit, I then mark it with a number hence making the results correct so that when another patient comes in you will know that you have allocated this number to this person. Mark, age 50, Dunba Mission Hospital.

However, when our observations and conversations turned towards their work conditions, it became clear that testing one or a few clients at a time was a luxury few could afford. One tester, Cecilia, reported that in her clinic they administered one test at a time, but she recognized the impact of workload on misclassification errors in testing facilities with high volumes of clients:

We administer one test at a time […] we write down their names for us to tell which result belongs to each of them. I think that for those centers that have high volumes of clients, there is that chance to give a wrong test result if the strips are not properly labelled. I think the misclassification errors are made when issuing the results. Cecilia, age 38, Imchiliz Clinic.

The demand for rapid HIV testing, coupled with limited resources, contributed to high frequency testing, or testing of multiple people at once (batch testing). This was observed in many of the participating clinics. When asked to further elaborate on the impact of high client volumes, Karen and Mary explain how they, as HIV testers, often feel compelled to speed up the testing process (often to reduce waiting time), and acknowledge that this increase in frequency and pace may lead to improper labelling practices:

When you have tested a lot of people and you want to make your things [the testing process] come out quickly […] one may label wrongly, right. You may give someone a wrong test result. Karen, age 45, Avuma District Hospital.

Yah, it can occur maybe due to pressure. If the tester is not efficient enough to write down names or to put some symbols on the test kits to show which test belongs to each person, maybe he will end up giving the wrong result. Mary, age 35, Angrushi Clinic.

The workload associated with high frequency or batch testing is further compounded by repeat testing when obtaining discordant results. As discussed further in Skovdal et al. (2020) the rapid HIV test strategy in Zimbabwe stipulates that if the first test is positive, a different and second test needs to be run. If they produce discordant results, the two tests should be repeated. If the test results remain discordant, a third “tie-breaker” is to be run.

Yes, the workload may become too much since there will be many patients but especially when we experience a lot of discordant results plus that requires a lot of time of which you won’t be having such an amount of time to do everything perfectly. The patients will be standing outside in a long queue so for you to have such time to do all that, and you are the only tester with the different test kits revealing a different set of results. Mark, age 50, Dunba Mission Hospital.

Obtaining discordant results can be detrimental to the temporal flow of other activities, with repeat testing consuming additional time. The work environment spares little time to deal with discordant results. In these situations, Mark points to how HIV testers may not do everything perfectly, falling short of the precision assumed by the rapid HIV testing scripts.

The above accounts highlight recognition of a clash between two tempi: the desired and scripted tempo of rapid HIV test-kits, which assumes a balanced relationship between frequency and tempo, and the fast-paced tempo experienced at clinic-level, defined by high client volumes, repeat testing, and low staffing resources. This discrepancy may, as Karen, Mary and Mark suggest, result in diagnostic misclassifications.

### Mis*timing* affects the reading of test results

The scripted rapid HIV test practice stipulates that once a test-kit has been accurately labelled, an appropriate blood sample, in sufficient quantities, must be collected (see Table 1). This is done through a finger prick. The blood sample must then be added onto the test device, followed by a buffer mixture. As soon as the buffer mixture has been added, a waiting time ensues. This is the time required for the test-kits to produce a valid result. Confusingly, the different test-kits used in Zimbabwe have different scripted timings, or valid results intervals (VRIs). For one test-kit, the waiting time is 5–15 min, and the HIV tester is given strict instructions not to read results after 15 min. Two test-kits require a 15-min wait, yet for one of them the waiting time can be extended up to 60 min:

Wait a minimum of 15 min (up to 60 min) after buffer is added and read results. Test kit 1 standard operating procedures.

Read the test results 15 min after the addition of the Running Buffer. In some cases, a test line may appear in less than 15 min, however, 15 min are needed to report a non-reactive result. Read results in a well-lit area. Do not read results after 20 min. Test kit 2 standard operating procedure.

For both Test kits 1 and 2, the waiting times are 15 min, yet the VRIs differ significantly between the two tests. For Test kit 1, HIV testers have 45 min to read the test results. In contrast, for Test kit 2 testers only have five minutes. In the previous section we outlined the regularity of batch testing, yet none of our participants alluded to the fact that batch testing with Test kit 2—given the short window to read results—is in practice impossible to do. Although understanding of the required VRI may be lacking, there was general awareness about the need to wait 15 min before reading the results, as illustrated by Agnes:

If you do not give it enough time, the kit might give a negative result before it produces a positive result. You would have read the results too early before the positive line is produced. You’ll give the client a wrong result. Agnes, age 36, Lutu Mission Hospital.

This recognition and caution were however often undermined by work pressures, either a result from high workloads, related to high frequency testing, or general interruptions. Through our observations we noted that although the testers did often look at their watch when they had applied the buffer fluid (so at the start of the VRI), they did not typically set a timer to notify them of the end of the VRI. We also observed constant distractions during the wait time, phone calls, colleagues knocking on the door, and clients popping in, making it difficult for testers to stick to the VRI, even if they were timed and only did one test at a time. These everyday issues were further exacerbated by different ‘crisis moments’, such as the long queues noted by Mark above, or stress induced by clients who expressed impatience:

Also, sometimes the clients can hustle you up. They won’t be patient enough so you will be pushed to give the results maybe earlier than the required time. Mary, age 35, Angrushi Clinic.

In our observations we witnessed multiple incidents of impatient clients who directly confronted HIV testers to vent their frustrations. In Kuvubam Polyclinic, one incident provides insight into why HIV testers may want to speed up the testing process:

Mbuya carried out the test on him. It was a very tense session because he was showing signs of being violent and he was extremely agitated. Even the neighbour pointed out that since morning he had become very short tempered and annoyed very easily. When Mbuya pricked him, he almost slapped her. Luckily the neighbour was there to restrain him. The client was extremely annoyed, and it was written all over his face. Participant observation, Kuvubam Polyclinic, 26.04.2018.

These accounts and observations highlight circumstances that may result in premature readings of test-kits. They indicate a different form of tempi clash. The tempo at which the test results are produced is determined by the timing specifications of the given test device. These timing specifications, relating to the VRIs, are considered stable and objective times that must be followed. The rapid HIV testing script therefore assumes that HIV testers synchronize and pace their activities of testing to fit with the objective timing criteria inscribed in the rapid HIV testing technology. However, as the examples by Rose and Mary indicate, this synchronization is not always possible, either because of high frequency testing or the influence of impatient clients. The latter example adds a dimension to the tempi clash, as HIV testers must juggle and synchronise not only the objective timing criteria of the test-kits, but also the subjectivity of time as experienced by clients who are anxiously waiting for their results. As Adam [19] argues, times of stress may be perceived to go faster than they do in reality. Likewise, when one is struck with boredom, it feels like time slows down. The quote by Mary points to this subjectivity of time, suggesting that HIV testers may be ‘pushed’ into reading test results prematurely, increasing the chances of mistiming the reading of test results.

### Recording is time-consuming, but prioritized, affecting *sequential* steps

Recording requires that the HIV testers write down the test results and client information in multiple books and registers. This recording was considered an important but strenuous and time-consuming process:

Recording is time-consuming. It is very time-consuming. To avoid leaving out one of those books [where test results are registered] you should have a sequence that you follow after testing a client i.e., [first noting results in] the pre-ART register, attendance register, dispensing register, pharmacy register and then allocate a green book. Cecilia, age 38, Imchiliz Clinic.

Cecilia’s list of various registers demonstrates why recording is considered a time-consuming activity, taking up much of the HIV testers’ time. Despite the time-consuming character of the work, recording was still regarded as an essential part of the rapid HIV testing script:

There is no other way out; we must record the details because they are important to us as a facility, so they have to be recorded at all times. It does not matter what the workload is like, recording must be done at all times. Tino, age 28, Lindase Mission Hospital.

The recording of test results was thus said to be done in meticulous detail. Only a few participants could give examples of HIV test results not being adequately recorded in the required registers:

We might see that there is a client who tested positive, and we ask whether she refused to be on ART because her details won’t be recorded in the other register. We will realize that it was a mistake. If there is anything that can be corrected, we will correct it so that the information recorded in all the books will be the same. Cecilia, age 38, Imchiliz Clinic.

Recording test results is a prioritized step of the HIV testing script. However, the time-consuming nature of this prioritized step, coupled with high frequency testing, may have contributed to a de-prioritization of other steps in the finite timeframe of “rapid” HIV testing, such as reading test results before they are ready or by omitting counselling, as explained by Alice:

If there are many clients to attend to, sometimes you don’t have enough time to counsel people. […] If there are many people, it means that the level of counselling may become poor because you will be having a lot of clients. Alice, age 41, Ango District Hospital.

Also our observations documented instances of counselling being omitted due to workloads and time pressures. To maintain the momentum achieved by testing multiple people at once, an HIV tester at Lutu Mission Hospital was observed to provide counselling to multiple clients at once, in a single session. These findings suggest that following some aspects of the script (e.g., recording) comes at a cost to the time available for other steps in the testing process.

## Discussion

With a focus on the scripted and practiced tempo of rapid HIV test labelling, reading, and recording, we set out to examine the factors that establish discrepant temporal flows between intended and actual testing practices, potentially contributing to diagnostic misclassification errors.

Our main finding is that there are substantial temporal dissonances between the assumed time available for scripted rapid HIV testing and the frequency and volume of tests carried out in real-life settings. The temporal flows inscribed in the rapid HIV testing script assume a calm and steady environment, which allows for precise and predictable testing practices. Yet, our analysis of everyday rapid HIV testing practices reveal that high client volumes, the frequent need for repeat testing, low staffing levels, and impatient clients contribute to fast-paced testing practices, characterized by a range of different short-cuts—or domestications [21] of the rapid HIV testing script to fit local realities—that heighten the risk of misdiagnosis. Specifically, we found quality compromises related to labelling errors when testing multiple clients at once, reading of test results too early or too late, and deprioritizing certain sequential steps. These findings point to how the very quality of testing practices depends on availability of time, a resource, which in these high frequency testing facilities, was both observed and considered by the rapid HIV testers themselves to be a scarce resource, an argument we have alluded to elsewhere [14]. Some of these qualitative insights, particularly those related to the premature reading of results, both corroborate and explain findings from our quantitative investigation. Here we introduced a Smartphone camera application in some health settings to capture the time elapsed between test initiation and reading of results. Out of 76 observations, there were 41 instances (53.9%) of premature reading of test results [6].

Our qualitative findings go some way to explain why 90% of rapid HIV tests observed by Wolpaw et al. [15] in South Africa were mistimed, and support previous research documenting time-constraints as a contributor to quality compromises [5] and potential misdiagnoses [4, 14] in rapid HIV testing. However, our findings go a step further. They show that errors are essentially embedded in the design of the testing programme from the outset, by inscribing assumptions around the calm, careful and timely way rapid HIV testing can be done. While work on mistiming of rapid HIV tests is important, its focus on user errors may easily lead to solutions that propose ever-increasing monitoring and surveillance of testers (with a danger to slide into punitive measures e.g., if used for staff performance reviews). This ignores the fact that—as our findings show—the testers we interviewed were acutely aware of the ways in which time-pressures negatively impacted the quality of their work, and as we report elsewhere, devised strategies to try to overcome some of the pressures that negatively affected the quality of their testing [22]. Rather than irresponsibly deviating from the testing script, they were trying—and sometimes failing—to perform as well as they could under the difficult circumstances. Moreover, what further challenges their efforts is a general invisibility of misdiagnosis. As we discuss elsewhere [18], rapid HIV testers have no real way of ascertaining the accuracy of their test results. When they in roundabout, and often coincidental ways discover a misdiagnosis, it is attributed to mislabeling and poor compliance with the test strategy [18], all ‘crisis-points’ that this paper show have temporal root causes.

These observations resonate with the work of Beisel et al. [23], who, from qualitative research on malaria rapid diagnostic test use in Uganda, Tanzania and Sierra Leone, also find a dissonance between the time inscribed into the tests and the high workload and low staffing realities characteristic of their study settings. They too note how staff must adapt and domesticate time-consuming rapid malaria test kits to fit with local realities. Together, our study findings challenge the aspiration of diagnostic test technologies that can be seamlessly introduced at the point-of-care anywhere in the world. Beisel and colleagues [23] argue that rather than being surprised by the tinkering with and short-cuts done by testers at the point-of-care, one should be surprised at the assumptions embedded in the technology instead. Following this argument, the problem does not lie with the users but with the design aspiration to make rapid diagnostic tests universal, and for assuming a good fit with resource-constrained health settings.

A few methodological constraints deserve to be mentioned. First, our design is prone to social desirability bias. To safeguard their personal or their organization's reputation, some individuals may have performed or portrayed themselves in a particularly good light. Relatedly, a selection bias could have been introduced by the Matrons or Sisters who acted as gatekeepers. However, by including participant observations in our methods; being in the testing rooms and spending time in the clinics, observing workflows and staff interactions, we have tried to preempt and limit the influence of selection bias. Second, and linked to this, our cross-sectional and rapid ethnographic approach meant we likely only observed more performative practices and were unable to capture changes over time. Nonetheless, our study points to the benefits of temporal analyses. Not only to understand how time affects rapid HIV testing practices and local domestication processes, but also to shift focus and blame away from the testers to the socio-material context, with errors being embedded into the rapid diagnostic test design and HIV testing programme set up. While this study specifically examines rapid HIV testing, applying a temporal lens to other areas of point-of-care testing, such as COVID-19 and malaria, may be beneficial.

## Conclusion

We have uncovered time-dilemmas and substantial discrepancies between the temporal flows assumed and inscribed into rapid HIV test kits (their intended use) and those presented by the high frequency testing and low resource and staffing realities of healthcare settings in Zimbabwe—revealing temporal root causes of misdiagnosis. Whilst rapid HIV tests may be quick from the perspective that it is now possible to circumvent exceedingly slow laboratory HIV testing, these findings indicate that their rapidity is contingent on a slow, steady, and controlled environment—adding a different dimension to the meaning of “rapid” HIV testing. Moreover, our findings demonstrate the potential of temporal analyses to uncover difficulties in attuning rapid diagnostic test technologies to local contexts. Such analysis may help identify potential misdiagnosis ‘crisis points’ in point-of-care testing and the need for public health initiatives that challenge the underlying temporal root causes of misdiagnosis. This may include specifying a maximum number of rapid diagnostic HIV tests a tester can safely perform per hour/day, given the test kit SOPs. This may force programme planners to budget for adequate staffing levels and could also be used in monitoring and evaluation systems to alert the relevant authorities when staffing levels do not allow for a safe testing frequency (e.g., by noting testing frequency and actual staff ratios). In addition to improved staffing, programme planners should be encouraged to re-think processes or explore the value of certain technologies—such as electronic medical records—in saving time and minimizing errors.

This paper highlights that understanding the complex relationship between technologies that travel, time and practice is vital to successfully address and prevent misdiagnosis in future HIV testing programmes.

## Data Availability

Data used for the study are available from the corresponding author on reasonable request.
